# Colic in Horses

**Published:** 1880-07

**Authors:** Geo. H. Berns

**Affiliations:** Professor of Equine Practice; Consulting Surgeon of Columbia Verterinary College; Brooklyn, N. Y.


### ART. XIV.—COLIC IN HORSES.

PART OF A CLINICAL LECTURE RECENTLY DELIVERED AT COLUMBIA VETERINARY COLLEGE.

BY GEO. H. BERNS, D.V.S.,

PROFESSOR OF EQUINE PRACTICE; CONSULTING SURGEON OF COLUMBIA VERTERINARY COLLEGE.

BEFORE we conclude the diseases of the digestive organs, I wish to call your special attention to one of the most prevalent, and therefore most important, diseases of equine practice, namely, *colic in its various forms.*

When we consider the injudicious management that our horses frequently receive, together with the peculiar anatomical arrangement of the cardiac opening of the stomach, which prevents a horse from vomiting when his stomach is overloaded, or contains indigestible food, it is not at all surprising that this form of trouble should be so very frequent. When man, the most intelligent of all the animal creation, will not infrequently fill his stomach with some favorite article of food to that extent as to compel nature to throw it off, by causing the individual to vomit, is it to be wondered at that a horse, after being kept in harness for a number of hours, and deprived of food and water for an inordinate length of time, will, on being put in front of a full manger, greedily swallow six or eight quarts of oats without masticating and salivating it properly, or gulp down too large a quantity of cold water? I do not think it is.

The majority of cases of colic are, in my opinion, due to the injudicious management of horses on the part of their owners or attendants, and could be readily avoided if sufficient judgment and care were exercised.

For all practical purposes, it will be sufficient for us to consider three different and distinct diseased conditions, under the head of colic:

1st. Spasmodic colic.

2d. Flatulent colic.

3d. Inflammation of the stomach or bowels.

These three forms of disease present a certain train of symptoms in common, and certain other symptoms which are characteristic, and therefore diagnostic of each particular form.

Spasmodic colic is a spasmodic contraction of the muscular coat of some portion of the intestines, due in the majority of cases to some substance acting as an irritant in the alimentary canal; in some instances, it may be due to reflex action. The muscular coat, which is composed of circular and longitudinal involuntary muscular fibres, is liable to spasms or cramps; and, when this takes place, the intestinal tract becomes locally contracted to that degree that the alimentary matters are, at the place of spasm, arrested in their course, causing, while the cramp continues, pain of a very severe character.

The causes of spasmodic colic are improper food, sudden changes of diet, inordinate quantities of food, particularly when given after long fasting, when an animal is very apt to bolt it down without properly preparing it for stomach and intestinal digestion; a draught of cold air, or a drink of cold water, when a horse is bathed in perspiration; washing a horse's legs or belly with a hose when he is sweating, etc. All these causes can be avoided, if proper care is taken; but there are numerous other causes which occasionally produce spasmodic colic. Some horses are particularly prone to it, and I look upon these cases as being caused, in many instances, by acidity of the stomach, or an irritable condition of the intestinal tract. The least little trivial circumstance which, in hundreds of horses, would not cause any disturbance at all, is often productive of violent spasms of the bowels, particularly in loosely-coupled, flat-sided animals.

Again, parasites, intestinal concretions, mesenteric abscesses, etc., may occasionally produce colic. I remember seeing a horse in the hospital of the New York College of Veterinary Surgeons some years ago which would manifest violent colicky pains after exercise, but while in the stable he appeared to be in the best of health. Examination per rectum of the bladder and that portion of the intestinal canal within reach, revealed nothing abnormal. I am under the impression, however, that this was a case of intestinal calculi. Unfortunately, I lost sight of the horse when he was removed from the hospital, and never learned how the case terminated.

*Symptoms.*—The horse is suddenly seized with a severe pain in the belly. He paws, stamps, and strikes his belly with his hind feet. He may throw himself down violently, issuing a kind of a grunt from the fall, roll upon his back, kick, crouch all into a heap, suddenly jump up, shake himself, and appear relieved; but for a short time only. He again begins to paw, looks at his flank, and probably rubs his sides and neck against the partition of his stall, passes small quantities of fæces, and sometimes urine; at other times frequent attempts at urinating are made, but prove unsuccessful, from the fact that the neck of the bladder is spasmodically contracted. Each successive paroxysm generally returns but to be more violent and of longer duration than the preceding one, and if a case of this description is not relieved, the intervals of ease generally become so short as to be hardly noticeable. The patient breaks out into a profuse perspiration, drops of sweat are trickling from his brows and eyelashes, he becomes delirious, his eyes look wild and frantic, he is heedless of all surroundings, cold sweats bedew his body, his ears and legs become deadly cold, he falls, or throws himself down, tremors follow, and death ends his sufferings. At the onset of the disease, and during the intervals of rest, the pulse is but little altered, but during the paroxysms it is increased in frequency, and as the disease advances it becomes accelerated and feeble, the conjunctiva and Schneiderian membranes become red, and the breathing short and quick. Examination per rectum will sometimes reveal a disturbed condition of the bladder, due to spasm of the sphincter. In some instances, more especially when the animal has been fed on grass, or raw potatoes, etc., there is diarrhœa, and an escape of much fœtid flatus.

*Treatment.*—The treatment consists in the administration of anodynes, antispasmodics, cathartics, and enemas. Carefully inquire into the history of the case, and ascertain if possible the cause of the attack, then regulate your treatment accordingly. If the spasm be due to improper or an inordinate quantity of food greedily devoured, it is your best course to combine a cathartic with the anodyne and antispasmodic in order to aid in the elimination of the offending substance from the system. In such cases warm water enemas also prove very serviceable, tr. of opium, sulphuric ether in ½ oz. doses, combined with from 4 to 8 drachms of aloes in sol.—or the compound spirits of ether co nbined with opium, and a cathartic are the medicinal agents that may be employed. If the cramp be due to a drink of cold water, a draught of cold air or any other external cause, that does not act directly upon the parts involved, the spasm of the bowels must be reflex, and in my opinion it is folly to order cathartics in these cases. Antispasmodics and anodynes will generally relieve the patient permanently. I know of no drench, that will relieve a case of spasmodic colic quicker and more effectually than the following, for the formula of which I am indebted to one of my colleagues.

*Recipe,* Tr. opi, 1½ oz.

Æther sulphuric, 1½ oz.

Spir. camphor, ½ oz.

Fl. extr. bellad., 1 drachm.

Olei lini., ½ pint.

This drench may be repeated every hour if necessary, till relief. The after-treatment consists in abstaining from food and water for some hours after the acute symptoms have passed off. If you have reasons to suspect intestinal concretions full doses of some active cathartic should be given at intervals of four or five days. If you have parasites as the cause of colic, anthelmintics should be employed. In acidity of the stomach and an irritable condition of the bowels, antacids and careful dieting are to be recommended, vegetable or mineral tonics are sometimes called for to improve the general health of patient.

At our next lecture the subject of flatulent colic will be considered.

